# Effect of isometric exercise on blood pressure in prehypertensive and hypertensive individuals: protocol for a systematic review and meta-analysis of randomized controlled trials

**DOI:** 10.1186/s13643-022-01974-9

**Published:** 2022-05-20

**Authors:** Patrícia Caetano de Oliveira, Alexandre M. Lehnen, Gustavo Waclawovsky

**Affiliations:** Instituto de Cardiologia do Rio Grande do Sul/Fundação Universitária de Cardiologia, Av. Princesa Isabel, 395 Santana, Porto Alegre, RS 90620-001 Brazil

**Keywords:** Prehypertension, Hypertension, Blood pressure, Isometric exercise

## Abstract

**Background:**

Systemic arterial hypertension (HTN) is the leading risk factor of cardiovascular disease death. Lifestyle changes are key for the prevention and management of HTN. Regular aerobic exercise training is recommended as part of the management of HTN, and dynamic resistance exercise should be prescribed as an adjuvant to aerobic training. Recent evidence points to the potential benefits of isometric resistance training in reducing blood pressure (BP). Yet, the hypotensive effect of isometric exercise in prehypertensive and hypertensive individuals is not fully understood. Thus, we will examine the effect of isometric exercise in prehypertensive and hypertensive individuals through a systematic review and meta-analysis.

**Methods:**

Our systematic review study will include randomized controlled trials (RCTs) selected from the electronic databases MEDLINE (PubMed), Cochrane, LILACS, EMBASE, Web of Science, and PEDro published in English, Spanish, and Portuguese languages. We will follow the PRISMA-P (Preferred Reporting Items for Systematic Review and Meta-Analysis Protocols) and PICOS framework. Our search will involve studies with both male and female participants aged 18 years or more diagnosed with prehypertension or HTN performing one session of isometric exercise (acute effect) or isometric exercise training (chronic effect) compared to a control group (no exercise). We will use the Cochrane Risk of Bias 2 (RoB 2) tool to evaluate the quality of the studies and RStudio software (v1.3.959 for Windows) for statistical analyses.

**Discussion:**

A meta-analysis of a homogeneous sample of prehypertensive and hypertensive individuals involving isometric handgrip exercise alone can further support previous findings and improve our understanding and recommendations for the management of these populations.

**Systematic review registration:**

PROSPERO CRD42020213081.

**Supplementary Information:**

The online version contains supplementary material available at 10.1186/s13643-022-01974-9.

## Background

Systemic arterial hypertension (HTN) prevalence is estimated at 30% of the worldwide population, affecting nearly 1.4 billion adults [1, 2]. The World Health Organization (WHO) has projected this number will escalate to 1.6 billion people by 2025 [1, 3]. This is a concerning scenario because a 10-mmHg increase in systolic blood pressure (SBP)/diastolic blood pressure (DBP) levels has been strongly associated with an increase in the rate of ischemic heart disease deaths [4, 5]. HTN represents the leading risk factor for cardiovascular diseases (13%) as compared to other risk factors including smoking (9%), high blood glucose (6%), physical inactivity (6%), and excess body weight (5%) [6].

The practice of regular aerobic exercise has been traditionally recommended as the first-line nonpharmacological management of cardiovascular diseases. Dynamic resistance exercise involving joint movement is recommended as an adjuvant intervention to aerobic training [7–9]. Interestingly, isometric resistance exercise (not involving joint movement) is apparently effective in reducing blood pressure (BP) levels as evidenced in meta-analyses published over the last 10 years [10–16]. A meta-analysis by Cornelissen et al. [12] evaluated the hypotensive effect of aerobic, dynamic resistance, and isometric resistance training. They found that isometric training elicited BP reductions greater than those seen in the control group and with other exercise modalities as well [12]. Yet, this meta-analysis included only four studies of isometric training.

Besides the small number of randomized controlled trials (RCTs) included, the samples of participants from the studies included in these meta-analyses are not fully representative of prehypertensive or hypertensive populations [10–12, 17]. They are usually heterogeneous samples of normotensive, prehypertensive, and/or hypertensive individuals, making it difficult to generalize the results for each group. Moreover, some studies involved participants with other comorbidities that might have affected their results. It is crucial to examine restricted samples of hypertensive individuals as they show higher BP levels in response to exercise and require longer recovery intervals between resistance exercise sets compared to normotensive individuals [18].

In addition, most meta-analyses [10, 12, 13, 16, 17] evaluated the effect of isometric exercise from studies with interventions of combined exercise involving upper and lower limbs, and since the amount of active muscle mass involved is different, hemodynamic responses to this type of exercise can be also different [19, 20]. To our understanding, if handgrip exercise alone is proven effective for the prevention and management of HTN, it could be prescribed as an alternative exercise approach for those who are not able to perform isometric leg exercise due to physical limitation, peripheral artery disease, or any other limiting condition. Although recently published meta-analyses [14, 15] showed good methodological quality, Jin et al. [14] evaluated a small number of studies (only seven) in their meta-analysis (four with hypertensive individuals, two with normotensive individuals, and one with prehypertensive individuals). Loaiza et al. [15] conducted a more consistent metanalysis with 11 articles of prehypertensive and hypertensive individuals; yet, three RCTs [21–23] did not use a control group for comparison of the analyses.

Because of the lack of robust metanalysis evidence, only the American College of Cardiology/American Heart Association Task Force on Clinical Practice Guidelines included isometric resistance exercise as a nonpharmacological intervention for the prevention and management of hypertension [8]. The European Association of Preventive Cardiology (EAPC) and the European Society of Cardiology (ESC) Council on Hypertension [24] pointed out that both dynamic and isometric resistance training can be recommended for secondary prevention in individuals with HTN, but not in those with pre-HTN. Both Canadian [25] and Brazilian [26] guidelines do not mention isometric exercise as either an adjuvant or alternative approach for the management of HTN. In addition, it has been proposed to make a distinction of populations for improved recommendations: (1) individuals with normal BP, but with risk factors for HTN; (2) individuals with normal-to-high BP; and (3) individuals with HTN.

Thus, given the importance of controlling and maintaining BP within normal levels throughout exercise and exercise training, and to provide the best evidence to inform guideline recommendations, it is valuable to conduct a consistent meta-analysis with a special focus on prehypertensive and hypertensive populations. To the best of our knowledge, there is no meta-analysis of studies investigating the acute effect of handgrip exercise on BP and they have examined only the chronic effect of this type of exercise. Hence, we developed a study protocol for a systematic review and meta-analysis including RCTs evaluating the BP-reducing effect of isometric exercise or training in prehypertensive or hypertensive individuals.

## Methods

**Fig. 1:**
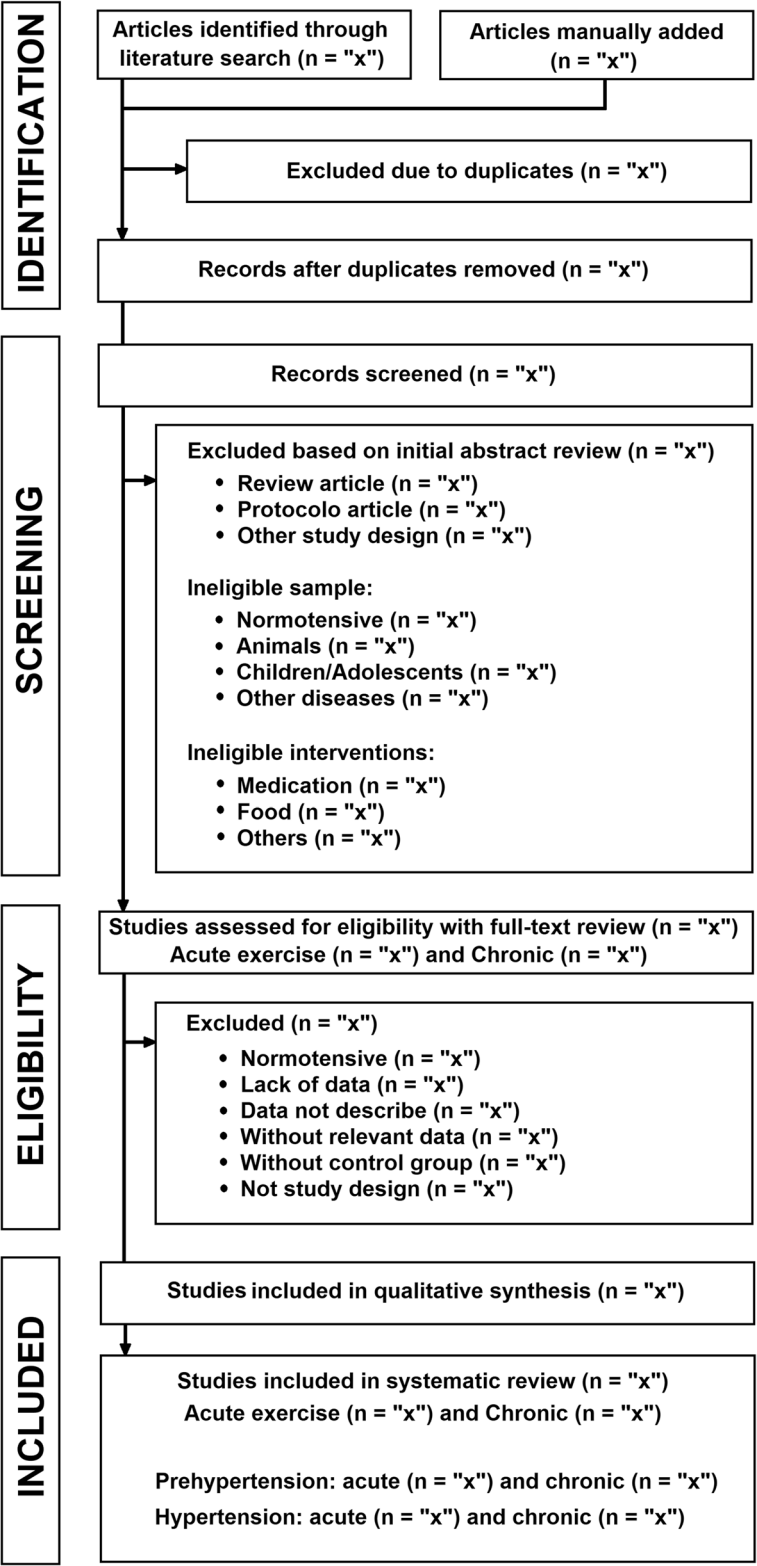
Flowchart of the systematic review study

The study protocol was developed based on PRISMA-P (Preferred Reporting Items for Systematic Review and Meta-Analysis Protocols) 2015 [27] and Cochrane systematic review methodology [28]. The protocol for the systematic review and meta-analysis was registered in the International Prospective Register of Systematic *Reviews* (PROSPERO) (“www.crd.york.ac.uk/PROSPERO/”, “CRD42020213081” and registration date 09/10/2020). Figure 1 summarizes the study design.

### Eligibility criteria

The PICOS framework was used to formulate eligibility criteria, as follows: *Population* (individuals aged ≥ 18 years diagnosed with pre-HTN or HTN); *Intervention* (one session of isometric resistance exercise or isometric handgrip training); *Comparison* (isometric resistance exercise or training versus [no exercise or training] control group); *Outcome* (SBP and DBP measurements by auscultation, oscillometry or 24-h ambulatory BP monitoring); *Study* (RCTs).

Inclusion criteria for this review study include “prehypertension” and/or “hypertension” in the title and/or abstract used to describe the main characteristic of the study sample. The main body of the article must have a clear description of the study population (adults) and diagnosis of HTN: sustained high BP levels and/or use of antihypertensive drugs together with a medical history and/or skilled medical assessment. The criteria for “intervention” include a study with two or more arms, either isometric resistance exercise or training using handgrip as “comparator” versus a control group (no session of exercise or physical training, or receiving only primary care, usual care, care while on a waiting list etc.). For the purpose of conducting subgroup analyses, only RCTs of prehypertensive or hypertensive individuals, defined in each RCT (overall, sustained high BP levels and/or use of antihypertensive drugs together with a medical history and/or skilled medical assessment), will be selected for the meta-analysis.

### Inclusion and exclusion criteria

We will select all studies of individuals aged 18 or more with HTN measured using auscultation, oscillometry, or 24-h ambulatory BP monitoring (ABPM) involving a single session of isometric handgrip exercise (acute effect or PEH) or isometric handgrip exercise training (chronic effect) as the intervention. The following FITT (frequency, intensity, type, and time of exercise) principles will be applied for a single session intervention: *Frequency*, a single session; *Intensity*, no limitation; *Type*, isometric handgrip exercise; *Time*, no limitation, and for studies involving exercise training: *Frequency*, at least two times per week; *Intensity*, no limitation; *Type*, only sessions of isometric handgrip exercise; *Time*, no time limitation per session and minimum duration of 4 weeks.

Studies involving other interventions associated with exercise but with a clearly defined isometric resistance group and a control group (no exercise) will be fully reviewed for inclusion. Studies with participants taking antihypertensive medications will be eligible when medication was introduced either ≥ 4 weeks before the exercise/training intervention or during the intervention and data analysis.

Studies with individuals taking medication other than antihypertensive drugs or concomitant dietary (or supplement) interventions as well as review studies, studies with animal experimentation, and studies that involve any condition other than pre-HTN or HTN will be excluded. Studies with similar characteristics published in different journals will be carefully reviewed and excluded if considered “duplicated publication.”

### Search strategy

A prior search was conducted in the MEDLINE database via PubMed to ascertain whether the research question of our review meets the FINER criteria (feasible, interesting, novel, ethical and relevant). A search strategy for RCTs was then developed to be undertaken by two independent reviewers (PCO and GW) in the databases recommended in the Cochrane Handbook for Systematic Reviews of Interventions [28]: MEDLINE, EMBASE (database of published European literature), Web of Science, and Cochrane databases (for access to trials that may not be indexed in MEDLINE and EMBASE). To broaden our search results, we will conduct searches for the Latin American literature through LILACS (Latin American and Caribbean Health Sciences Literature/Virtual Health Library [VHL]). Given that the intervention of interest may also be part of physical therapy rehabilitation programs, our searches will also be conducted in the Physiotherapy Evidence Database (PEDro). To minimize any publication bias, searches will also be undertaken on online gray literature including OpenGrey and the Brazilian Coordination for the Improvement of Higher Education Personnel (CAPES) Bank of Theses and Dissertations. For unpublished ongoing trials, our searches will be undertaken in clinical trial registries: Brazilian Clinical Trials Registry (ReBEC), Clinical Trial.gov, and WHO International Clinical Trials Registry Platform (ICTRP). We will gather data through a careful review of the articles retrieved and will contact authors by email to obtain any additional information as required. Articles in Portuguese, English, and Spanish languages with no date of publication limits will be eligible for inclusion. Upon completion of the review, we will undertake an additional search of all databases and registry platforms to ensure the inclusion of the most recent studies.

The main search terms will include “hypertension,” “exercise,” “isometric exercise,” and “blood pressure” (Table 1). The search terms for the study design (randomized clinical trial, RCT) will be entered in the databases MEDLINE [29] and EMBASE [30] for more accurate and sensitive searches (Table 1). The two study reviewers (PCO and GW) will work independently selecting the studies after an initial screening of titles and abstracts. When there will be no sufficient information in the abstract, the reviewers will retrieve and read the full text of the article. Any discrepancies will be resolved through a consensus discussion and, if necessary, any disagreements on the inclusion criteria will be resolved by a third reviewer (AML).

**Table 1:** Search terms to be used to search MEDLINE, EMBASE, Web of Science, Cochrane, LILACS, and PEDro databases

**MEDLINE (PubMed)**	
***Hypertension:*** *(blood pressure, high OR blood pressures, high OR high blood pressure OR high blood pressures OR pre hypertension OR prehypertension OR pre hypertension OR prehypertension) AND* ***Exercise:*** *(exercise OR exercises, isometric OR isometric exercises OR warm-up exercise OR exercise, warm-up OR exercises, warm-up OR warm up exercise OR warm-up exercises OR exercise, aerobic OR aerobic exercises OR exercises, aerobic OR aerobic exercise OR endurance, physical OR endurance, physical OR physical endurance OR training, resistance OR strength training OR training, strength OR weight-lifting OR strengthening program OR strengthening program, weight-lifting OR strengthening programs, weight-lifting OR weight lifting strengthening program OR weight-lifting strengthening programs OR weight-lifting exercise program OR exercise program, weight-lifting OR exercise programs, weight-lifting OR weight lifting exercise program OR weight-lifting exercise programs OR weight-bearing strengthening program OR strengthening program, weight-bearing OR strengthening programs, weight-bearing OR weight bearing strengthening program OR weight-bearing strengthening programs OR weight-bearing exercise program OR exercise program, weight-bearing OR exercise programs, weight-bearing OR weight bearing exercise program OR weight-bearing exercise programs OR activities, motor OR activity, motor OR motor activities OR physical activity OR activities, physical OR activity, physical OR physical activities OR locomotor activity OR activities, locomotor OR activity, locomotor OR locomotor activities) AND* ***Blood Pressure:*** *(Pressure, Blood OR Diastolic Pressure OR Pressure, Diastolic OR Systolic Pressure OR Pressure, Systolic OR Pressures, Systolic OR Arterial Pressures OR Pressure, Arterial OR Pressures, Arterial OR Arterial Tension OR Arterial Tensions OR Tension, Arterial OR Tensions, Arterial OR Blood Pressure, Arterial OR Arterial Blood Pressure OR Arterial Blood Pressures OR Blood Pressures, Arterial OR Pressure, Arterial Blood OR Pressures, Arterial Blood OR Ambulatory Blood Pressure Monitoring OR Monitoring, Ambulatory Blood Pressure OR Blood Pressure Monitoring) AND* ***Type of study:*** (Randomized controlled trial[pt] OR controlled clinical trial[pt] OR randomized controlled trials[mh] OR random allocation[mh] OR double-blind method[mh] OR single-blind method[mh] OR clinical trial[pt] OR clinical trials[mh] OR (“clinical trial”[tw]) OR ((singl*[tw] OR doubl*[tw] OR trebl*[tw] OR tripl*[tw]) AND (mask*[tw] OR blind*[tw])) OR (“latin square”[tw]) OR placebos[mh] OR placebo*[tw] OR random*[tw] OR research design[mh:noexp] OR follow-up studies[mh] OR prospective studies[mh] OR cross-over studies[mh] OR control*[tw] OR prospectiv*[tw] OR volunteer*[tw])	
**EMBASE**	
***Hypertension:*** *('hypertension'/exp OR hypertension OR 'prehypertension') AND* ***Exercise:*** *('exercise' OR 'aerobic exercise' OR 'isometric exercise' OR 'warm-up exercise' OR 'physical endurance' OR 'endurance' OR 'training' OR 'resistance training' OR 'weight lifting' OR 'strengthening exercise' OR 'weight bearing' OR 'motor activity' OR 'physical activity') AND* ***Blood pressure:*** *(‘Blood Pressure’ OR ‘Diastolic Pressure’ OR ‘Systolic Pressure’ OR ‘Arterial Pressures’ OR ‘Arterial Tension’ OR ‘Arterial Blood Pressure’ OR ‘Ambulatory Blood Pressure Monitoring’ OR ‘Blood Pressure Monitoring’) AND* ***Type of study:*** *('randomized controlled trial' OR 'controlled clinical trial')*	
**Cochrane**	
***Hypertension:*** *(blood pressure, high OR blood pressures, high OR high blood pressure OR high blood pressures OR pre hypertension OR prehypertension OR pre hypertension OR prehypertension) AND* ***Exercise:*** *(exercise OR exercises, isometric OR isometric exercises OR warm-up exercise OR exercise, warm-up OR exercises, warm-up OR warm up exercise OR warm-up exercises OR exercise, aerobic OR aerobic exercises OR exercises, aerobic OR aerobic exercise OR endurance, physical OR endurance, physical OR physical endurance OR training, resistance OR strength training OR training, strength OR weight-lifting OR strengthening program OR strengthening program, weight-lifting OR strengthening programs, weight-lifting OR weight lifting strengthening program OR weight-lifting strengthening programs OR weight-lifting exercise program OR exercise program, weight-lifting OR exercise programs, weight-lifting OR weight lifting exercise program OR weight-lifting exercise programs OR weight-bearing strengthening program OR strengthening program, weight-bearing OR strengthening programs, weight-bearing OR weight bearing strengthening program OR weight-bearing strengthening programs OR weight-bearing exercise program OR exercise program, weight-bearing OR exercise programs, weight-bearing OR weight bearing exercise program OR weight-bearing exercise programs OR activities, motor OR activity, motor OR motor activities OR physical activity OR activities, physical OR activity, physical OR physical activities OR locomotor activity OR activities, locomotor OR activity, locomotor OR locomotor activities) AND* ***Blood Pressure:*** *(Pressure, Blood OR Diastolic Pressure OR Pressure, Diastolic OR Systolic Pressure OR Pressure, Systolic OR Pressures, Systolic OR Arterial Pressures OR Pressure, Arterial OR Pressures, Arterial OR Arterial Tension OR Arterial Tensions OR Tension, Arterial OR Tensions, Arterial OR Blood Pressure, Arterial OR Arterial Blood Pressure OR Arterial Blood Pressures OR Blood Pressures, Arterial OR Pressure, Arterial Blood OR Pressures, Arterial Blood OR Ambulatory Blood Pressure Monitoring OR Monitoring, Ambulatory Blood Pressure OR Blood Pressure Monitoring)*	
**LILACS**	
***LANGUAGE: Spanish*** ***Hipertensíon:*** *(tw:(Hipertensión)) OR (tw:(Prehipertensión)) AND* ***Ejercicio:*** *(tw:(Ejercicio Físico)) OR (tw:(Terapia por Ejercicio)) OR (tw:(Esfuerzo Físico)) OR (tw:(Aptitud Física)) OR (tw:(Actividad Motora)) AND (tw:(Presión Arterial)) AND* ***Presión arterial:*** *(tw:(Monitoreo Ambulatorio de la Presión Arterial))* ***LANGUAGE: Portuguese*** ***Hipertensão:*** *(tw:(hipertensão)) OR (tw:(Pré-Hipertensão)) AND* ***Exercício:*** *(tw:(Exercício Físico)) OR (tw:(Terapia por Exercício)) OR (tw:(Esforço Físico)) OR (tw:(Aptidão Física)) OR (tw:(Atividade Motora)) AND* ***Pressão arterial:*** *(tw:(pressão arterial)) OR (tw:(Monitorização Ambulatorial da Pressão Arterial))* ***LANGUAGE: English*** ***Hypertension:*** *(tw:(hypertension)) OR (tw:(Prehypertension)) AND* ***Exercise:*** *(tw:(exercise)) OR (tw:(Exercise Therapy)) OR (tw:(Physical Exertion)) OR (tw:(Physical Fitness)) OR (tw:(Motor Activity)) AND* ***Blood pressure:*** *(tw:(Arterial Pressure)) OR (tw:(Blood Pressure Monitoring, Ambulatory))*	
**PEDro**	
***Exercise:*** *Isometric exercise AND* ***Blood pressure:*** *blood pressure*	
**Web of Science**	
***Hypertension:*** *(hypertension or prehypertension) AND* ***Exercise:*** *isometric exercise AND* ***Blood pressure:*** *blood pressure*	
**Gray literature and unpublished studies**	
***OpenGrey:*** *isometric exercise AND blood pressure* Banco de Teses e Dissertações CAPES*: *exercício isométrico e pressão arterial em pré-hipertensos e hipertensos* ReBEC**: *pressão arterial (search 1), exercício e pressão arterial (search 2)* ClinicalTrials***: *hypertension OR prehypertension AND isometric exercise* ***WHO:*** *hypertension OR prehypertension AND isometric exercise* * Filter: Grande Área Conhecimento (CIÊNCIAS DA SAÚDE), Área Avaliação (EDUCAÇÃO FÍSICA, ENFERMAGEM, MEDICINA I, MEDICINA II, MEDICINA III, NUTRIÇÃO) ** Filter: tipo de estudo (intervencional), Situação de recrutamento (Recrutando, Recrutamento concluído, Análise de dados complete) *** Filter: Study type (interventional study (clinical trial))	

### Data extraction and management

After a complete search in each database, all articles retrieved will be exported as “.ris” or “.enib” files and imported into EndNote X9 arranged in folders by search platform, exclusion criteria, and eligibility. All duplicates will be removed using EndNote X9 *Find Duplicates* command (Menu *All references* / *References* / *Find duplicates*). Our reviewers (PCO and GW) will use a search tool (*Search*, *Title OR Abstract*) to check for any remaining duplicates. In the next step, they will read the full text of all eligible studies. If they are considered relevant, the main data of the studies selected will be extracted and compiled in a pre-structured database in Excel 2010 for Windows: (1) study (authors, journal, year of publication, intervention, characteristics of exercise session or training); (2) participants (age, gender, body mass index [BMI], medical condition, and antihypertensive drugs); and (3) methods (randomization, blinding) and outcome (sample size, mean, and standard deviation at baseline and post-exercise session or training). For eligible studies with results presented in graphs, we will contact the authors by email to obtain this data or use GetDate Graph Digitizer 2.26 to extract the data. For studies evaluating BP at different time points, we will independently compare baseline measurements with results at each time point. Then, our database will be formatted and imported into RStudio for data analyses.

### Risk of bias

The risk of bias in RCTs will be assessed using Cochrane RoB 2 tool included in the Cochrane Handbook [28, 31, 32]. The assessment is based on a set of six domains of bias, and judgement can be low, high, or unclear risk of bias: randomization sequence generation, allocation concealment, blinding of participants and personnel, blinding of outcome assessment, incomplete outcome data, and selective reporting. If participants are not blinded because blinding is not feasible for exercise intervention (one session of exercise or training), all studies will be classified as high risk of bias in the “blinding of participants and personnel” domain. No study will be excluded based on the risk of bias assessment. The risk of bias will be analyzed for the primary outcomes of interest in our review.

### Risk of overall bias in systematic reviews

We will evaluate the strength of the evidence using the Grading of Recommendations Assessment, Development and Evaluation tool (GRADE) [33]. This tool evaluates confidence in estimates of paired effects as well as classifies treatment in a network meta-analysis: study design, methodological limitations (risk of bias), inconsistency, indirectness of evidence, imprecision, publication bias, magnitude of effect, dose-response gradient, and residual confounders [33].

### Analysis strategy

We will conduct separate analyses for the acute effect of isometric resistance exercise and the chronic effect of isometric handgrip training. All measures of effect will be presented as mean differences (pre- and post-intervention and control) and their 95% confidence intervals (95% CIs) between exercise or training arms versus a (no exercise) control group. When data is reported in different units, the standardized mean difference will be used as it expresses the size of the intervention effect in each study relative to the variability observed in that study, together with their 95% CI. Since the standardized mean difference entails a broad interpretation of the results (in absolute units), we will convert these measures into proportions (%) so that the data are presented in a clear and more appropriate manner for the outcome of interest. Mean differences or standardized mean differences will be pooled using a fixed- or random-effects model which is appropriate. A random-effects model will be used if studies do not have sufficient similarities to warrant a fixed-effects model and/or the data is reported in different units.

The summary estimate and confidence interval of the random effect refer to the center of the distribution of intervention effects, but do not describe the width of the distribution. Since the confidence interval from random effects refers to uncertainty in the location of the mean of systematically different effects in the studies, we will consider calculated values for a prediction interval (variation in treatment effects over different settings including what treatment effect is to be expected in future patients) [34] (Chart 1S, supplementary material).

Heterogeneity (percentage of the variability in effect estimates) will be assessed using the *I*^2^ statistic and Cochran *Q* test for each pairwise comparison [28, 35] (Chart 2S; supplementary material). If heterogeneity is found (*p* < 0.05) for potential effect modifiers, it will be tested using subgroup analysis or meta-regression for potential effect modifiers with normal distribution using a quartile-quartile plot (qq-plot) and Shapiro-Wilk test [28]. If there will be high levels of heterogeneity but not enough data to explain them, we will not conduct a meta-analysis. Instead, we will present intervention effect estimates from individual studies.

Potential confounders including age, gender, BMI, intervention duration (minutes OR weeks), exercise frequency per week, intervention intensity (low, moderate, and high), and antihypertensive drug classes will be assessed a priori.

Considering that bias may occur from selective publication and/or suppression of results and consequently affect the validity of results, when appropriate, publication bias will be assessed using Egger’s test and a funnel plot [36]. If publication bias is detected (Egger’s test, *p* < 0.100), we will apply the “trim-and-fill” method to identify and correct the funnel plot’s asymmetry [37, 38].

In multi-arm trials, we will determine the intervention groups that are relevant to be included in the systematic review. If there are multiple intervention groups with similar categories, these groups will be pooled for a single pairwise comparison using the equations suggested by Cochrane [39] as follows:

- Sample size (*N*): *N*_group A_ + *N*_group B_;
- Mean (M): *N*_group A_ * *M*_group A_ + *N*_group B_ * *M*_group B_/*N*_group A_ + *N*_group B_;
- Standard deviation (SD): SD = √ (*N*_group A_ – 1) * SD^2^_group A_ + (*N*_group B_ – 1) * SD^2^_group B_ + (*N*_group A_ * *N*_group B_/*N*_group A_ + *N*_group B_) * (*M*_group A_ + *M*_group B_ – 2 * *M*_group A_ * *M*_group B_)/*N*_group A_ + *N*_group B_ – 1.

To avoid errors due to different data units in RCTs with multiple intervention arms and a single control group, the sample size for the control group will be weighted by the number of groups and participants included. For crossover RCTs, we will use a more conservative approach to determine the smallest unit error as proposed by Cochrane. Thus, we will collect all measurement data for exercise (pre- and post-intervention) and control sessions (pre- and post-control) and analyze them as parallel groups and individual SDs for each intervention.

In case of missing SDs for changes from baseline in any eligible study, they will be imputed from SDs for each time period (intervention and control) and we will calculate a correlation coefficient using the equations suggested by Cochrane as follows:

- Correlation coefficient (CC) = SD^2^_baseline_ + SD^2^_final_ − ∆ SD^2^/2 * SD^2^_baseline_ * SD^2^_final_
- ∆ SD = √ SD^2^_baseline_ + SD^2^_final_ – (2 * CC * SD_baseline_ * SD_final_).

If data are described as medians and upper and lower interquartiles in a given study, we will use the equation by Wan et al. [40] to determine the approximate values for the mean and SD.

All statistical tests will be two-tailed and the significance will be set at *p* < 0.05. Data modelizations will be performed with RStudio (version 1.3.959) using R package meta (version 3.6.1 for Windows). Chart 3S summarizes the main RStudio script (supplementary material) (database: META_ISOMETRICpas).

## Discussion and conclusions

A good number of studies have reported beneficial effects of isometric handgrip exercise on BP [10–16]. PEH > 2 mmHg in SBP is highly relevant for the management of HTN and is associated with a 14% reduction of the risk of mortality from stroke, 9% from coronary heart disease, and 7% in all-cause deaths [4, 41, 42]. However, there is no consensus in the literature regarding the expected magnitude of BP reduction in response to a single session of isometric exercise whether handgrip or any other type. Thus, there is no consensus in the literature regarding the expected magnitude of isometric exercise-induced PEH.

Yet, after a comprehensive evaluation of the published meta-analyses, we found that subgroup analyses are seldom conducted [10–12, 17], and when they are performed [13–16], some confounding effects are present such as combined upper- and lower-limb exercises [10, 12, 13, 16, 17] that may potentially interfere with hemodynamic responses [19]. We also found the analysis of heterogeneous samples consisting of individuals with pre-HTN and HTN. It is known that individuals with HTN have higher baseline BP compared to those with pre-HTN and the reduction in SBP/DBP may be of greater magnitude [43, 44] that they *respond* differently to the *stimulus* of *exercise* and need longer rest intervals between resistance exercise sets [18].

Bearing in mind the clinical significance of the hypotensive effect of isometric exercise training and the weakness of the studies found, we believe it requires further examination in prehypertensive and hypertensive individuals. It is also important to evaluate potential hypotensive effects of isometric handgrip or leg exercise alone (leg press, free squats, etc.) as some people are not able to perform certain types of exercises due to physical limitations or other reasons.

In conclusion, understanding the potential BP benefits of isometric exercise alone is important as it may provide alternative exercise options for the management of prehypertensive and hypertensive populations. A meta-analysis of a homogeneous sample of prehypertensive and hypertensive individuals involving isometric handgrip exercise (a single bout) or training can further support previous findings and improve our understanding and recommendations for the management of these populations.

## Supplementary Information

**Additional file 1: Chart 1S**. Formula for the prediction interval. **Chart 2S**. Formula and interpretation of heterogeneity in meta-analysis. **Chart 3S**. Main RStudio script. **Table 1S**. Description of articles that will be included in meta-analyses.

## Data Availability

Not applicable.

## Abbreviations

HTN: Systemic arterial hypertension
WHO: World Health Organization
SBP: Systolic blood pressure
DBP: Diastolic blood pressure
BP: Blood pressure
RCTs: Randomized controlled trials
PRISMA-P: Preferred Reporting Items for Systematic Review and Meta-Analysis Protocols
PICOS: Population, Intervention, Comparison, Outcome, and Study
ABPM: Ambulatory blood pressure monitoring
FINER: Feasible, Interesting, Novel, Ethical and Relevant
BMI: Body mass index
GRADE: Grading of Recommendations Assessment, Development and Evaluation
