# Supplementary material for: Effect of isometric exercise on blood pressure in prehypertensive and hypertensive individuals: protocol for a systematic review and meta-analysis of randomized controlled trials
Source: Syst Rev. 2022 May 20;11:100. doi: 10.1186/s13643-022-01974-9 (PMC9123753; doi:10.1186/s13643-022-01974-9)
Supplement: Supplementary file 1 — Additional file 1: Chart 1S. Formula for the prediction interval. Chart 2S. Formula and interpretation of heterogeneity in meta-analysis. Chart 3S. Main RStudio script. Table 1S. Description of articles that will be included in meta-analyses. [file 13643_2022_1974_MOESM1_ESM.docx]

Supplementary Material

**Effect of isometric exercise on blood pressure in pre-hypertensive and hypertensive individuals: protocol for a systematic review and meta-analysis of randomized clinical trials**

**Isometric exercise and blood pressure**

Patrícia Caetano de Oliveira^1^, Alexandre M Lehnen^1^, Gustavo Waclawovsky^1^

^1^ Instituto de Cardiologia do Rio Grande do Sul/Fundação Universitária de Cardiologia, Porto Alegre, Brazil.

**Corresponding author:**

Dr. Alexandre Machado Lehnen

Instituto de Cardiologia do Rio Grande do Sul/Fundação Universitária de Cardiologia

Av. Princesa Isabel, 395 Santana, 90620-001 Porto Alegre – RS Brazil

Phone:+55(51)32303600, branch 3636/3757

E-mail: amlehnen@gmail.com

**Chart 1S – Formula for the prediction interval**

| **Formula** |
| --- |
| 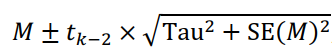 |
| Where M is the summary mean of a random-effects meta-analysis, t*_k_*_−2_ is the 95% percentile of a t distribution with k–2 degrees of freedom, *k* is the number of studies, *Tau^2^* is the estimated amount of heterogeneity and SE(M) is the standard error of the summary mean. |

**Chart 2S – Formula and interpretation of heterogeneity in meta-analysis**

| **Interpretation** | ***I*^2^ formula** |
| --- | --- |
| - **0% to 40%:** might not be important | 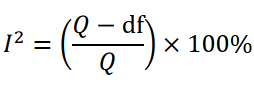 |
| - **30% to 60%:** may represent moderate heterogeneity* |  |
| - **50% to 90%:** may represent substantial heterogeneity* |  |
| - **75% to 100%:** considerable heterogeneity* |  |
| * The importance of the observed value of *I^2^* depends on the magnitude and direction of effects and strength of evidence for heterogeneity (confidence interval for *I^2^*: uncertainty of *I*^2^ is substantial when the number of studies is small). Q is the chi-squared statistic and *df* (degrees of freedom) is the degree of freedom. *I^2^* describes the percentage of variability in effect estimates that is due to heterogeneity. | |

**Chart 3S – Main RStudio script**

| 1. library (readxl) 2. META_ISOMETRICpas <- read_excel("META_ISOMETRICpas.xlsx") 3. View (META_ISOMETRICpas) 4. meta_HAS <- META_ISOMETRICpas <- metacont (t_n, t_mean, t_dp, c_n, c_mean, c_dp, Study, data = META_ISOMETRICpas, sm="MD") 5. meta_HAS 6. forest (meta_HAS, sortvar = Study, xlim = c(-15.0, 15.0), col.square = "blue", col.diamond = "red", digits = 1) 7. forest (meta_HAS, sortvar = Study, comb.fixed = FALSE,xlim = c(-15.0, 15.0), col.square = "blue", col.diamond = "red", predict= TRUE, digits = 1) 8. metabias (meta_HAS, method.bias = "linreg") 9. funnel(meta_HAS) 10. baujat (meta_HAS) 11. metainf (meta_HAS, pooled="random") 12. metareg (meta_HAS, ~SBP_baseline) 13. meta_HAS <- metareg (meta_HAS, ~SBP_baseline) 14. bubble (meta_HAS, col.line = "blue",col = "black") |
| --- |

**Table 1S – Description of articles that will be included in meta-analyses**

| **Meta-analysis references** | **Sample** | **RCTs included** | **Control group** | **Intervention group** |
| --- | --- | --- | --- | --- |
| Loaiza-Betancur et al. (2020)  Separate analysis for subgroups: pre-hypertensive and hypertensive individuals | Pre-hypertensive | Wiley, RL et al. (1992) | No exercise | Unilateral handgrip - dominant hand |
|  |  | Baross, AW et al. (2017) | No exercise | Bilateral handgrip |
|  |  | Goessler, KF et al. (2018) | No exercise | Bilateral handgrip |
|  |  | Gordon, BDH et al. (2018) | No exercise | Unilateral handgrip - dominant hand |
|  |  | Carlson, DJ et al. (2016) | Exercise at 5% MVC | Unilateral handgrip - dominant hand |
|  | Hypertensive | Pagonas, NJ et al. (2017) | Exercise at 5% MVC | Bilateral handgrip |
|  |  | McGowan, CL et al. (2007) | ---- | Unilateral vs. bilateral |
|  |  | Stiller-Moldovan, C et al. (2012) | No exercise | Bilateral handgrip |
|  |  | Farah, BQ et al. (2018) | No exercise | Bilateral handgrip |
|  |  | Millar, PJ et al. (2012) | No exercise | Unilateral handgrip - dominant hand |
|  |  | Taylor, AC et al (2003) | No exercise | Bilateral handgrip |
| López-Valenciano et al. (2019)  Separate analysis for subgroups: normotensive and “pre-hypertensive + hypertensive" individuals; included handgrip + leg exercise | Normotensive | Badrov, MB et al. (2013b) | No exercise | Unilateral handgrip - dominant hand |
|  |  | Millar, PJ et al. (2008) | No exercise | Bilateral handgrip |
|  |  | Ray, CA; Carrasco, DI (2000) | No exercise | Unilateral handgrip - dominant hand |
|  |  | Devereux, GR et al. (2011) | No exercise | Bilateral leg press |
|  |  | Gill, KF et al. (2015) | No exercise | Bilateral leg press |
|  |  | Howden, R et al. (2002) | No exercise | Bilateral leg press |
|  |  | Wiles, JD et al. (2010) | No exercise | Bilateral leg press |
|  |  | Wiles, JD et al. (2017) | No exercise | Free squats |
|  | Pre-hypertensive | Baross, AW et al. (2013) | No exercise | Bilateral leg press |
|  |  | Baross, AW et al. (2012) | No exercise | Bilateral leg press |
|  |  | Wiley, RL et al. (1992) | No exercise | Unilateral handgrip - dominant hand |
|  |  | Carlson, DJ et al. (2016) | Exercise at 5% MVC | Unilateral handgrip - dominant hand |
|  | Hypertensive | Pagonas, NJ et al. (2017) | Exercise at 5% MVC | Bilateral handgrip |
|  |  | Badrov, MB et al. (2013) | No exercise | Bilateral handgrip |
|  |  | Stiller-Moldovan, C et al. (2012) | No exercise | Bilateral handgrip |
|  |  | Taylor, AC et al (2003) | No exercise | Bilateral handgrip |
| Jin et al. (2017)  Separate analysis for subgroups: normotensive and “pre-hypertensive + hypertensive” individuals | Normotensive | Badrov, MB et al. (2013a) | No exercise | Unilateral handgrip - dominant hand |
|  |  | Badrov, MB et al. (2013b) | No exercise | Unilateral handgrip - dominant hand |
|  |  | Millar, PJ et al. (2008) | No exercise | Unilateral handgrip - dominant hand |
|  | Pre-hypertensive | Wiley, RL et al. (1992) | No exercise | Unilateral handgrip - dominant hand |
|  | Hypertensive | Stiller-Moldovan, C et al. (2012) | No exercise | Bilateral handgrip |
|  |  | Taylor, AC et al (2003) | No exercise | Bilateral handgrip |
|  |  | Badrov, MB et al. (2013) | No exercise | Bilateral handgrip |
| Inder et al. (2016)  Separate analysis for subgroups: normotensive and "pre-hypertensive + hypertensive" individuals; included handgrip + leg exercise | Normotensive | Badrov, MB et al. (2013a) | No exercise | Handgrip unilateral |
|  |  | Millar, PJ et al. (2008) | No exercise | Bilateral handgrip |
|  |  | Wiles, JD et al. (2010) | No exercise | Bilateral leg press |
|  |  | Devereux, GR et al. (2011) | No exercise | Bilateral leg press |
|  | Pre-hypertensive | Wiley, RL et al. (1992) | No exercise | Unilateral handgrip - dominant hand |
|  |  | Baross, AW et al. (2012) | No exercise | Bilateral leg press |
|  |  | Baross, AW et al. (2013) | No exercise | Bilateral leg press |
|  | Hypertensive | Taylor, AC et al (2003) | No exercise | Bilateral handgrip |
|  |  | Stiller-Moldovan, C et al. (2012) | No exercise | Bilateral handgrip |
|  |  | Badrov, MB et al. (2013) | No exercise | Bilateral handgrip |
| Carlson et al. (2014)  No separate analysis for subgroups | Normotensive | Badrov, MB et al. (2013a) | No exercise | Handgrip unilateral |
|  |  | Millar, PJ et al. (2008) | No exercise | Bilateral handgrip |
|  |  | Wiles, JD et al. (2010) | No exercise | Bilateral leg press |
|  |  | Devereux, GR et al. (2011) | No exercise | Bilateral leg press |
|  | Pre-hypertensive | Wiley, RL et al. (1992) | No exercise | Unilateral handgrip - dominant hand |
|  |  | Baross, AW et al. (2012) | No exercise | Bilateral leg press |
|  | Hypertensive | Badrov, MB et al. (2013) | No exercise | Bilateral handgrip |
|  |  | Stiller-Moldovan, C et al. (2012) | No exercise | Bilateral handgrip |
|  |  | Taylor, AC et al (2003) | No exercise | Bilateral handgrip |
| Cornelissen et al. (2013)  No separate analysis for subgroups | Normotensive | Millar, PJ et al. (2008) | No exercise | Bilateral handgrip |
|  |  | Wiles, JD et al. (2010) | No exercise | Bilateral leg press |
|  | Pre-hypertensive | Wiley, RL et al. (1992) | No exercise | Unilateral handgrip - dominant hand |
|  | Hypertensive | Taylor, AC et al (2003) | No exercise | Bilateral handgrip |
| Cornelissen et al. (2011)  No separate analysis for subgroups | Normotensive | Millar, PJ et al. (2008) | No exercise | Bilateral handgrip |
|  |  | Wiles, JD et al. (2010) | No exercise | Bilateral leg press |
|  | Pre-hypertensive | Wiley, RL et al. (1992) | No exercise | Unilateral handgrip - dominant hand |
|  | Hypertensive | Taylor, AC et al (2003) | No exercise | Bilateral handgrip |
| Owen et al. (2010)  No separate analysis for subgroups | Normotensive | Howden, R et al. (2002) | No exercise | Bilateral leg press |
|  |  | Wiley, RL et al. (2010) | No exercise | Bilateral leg press |
|  |  | Millar, PJ et al. (2008) | No exercise | Unilateral handgrip - dominant hand |
|  | Pre-hypertensive | Wiley, RL et al. (1992) | No exercise | Unilateral handgrip - dominant hand |
|  | Hypertensive | Taylor, AC et al (2003) | No exercise | Bilateral handgrip |

MVC: maximum voluntary contraction
